# Long-Term Aircraft Noise Exposure and Body Mass Index, Waist Circumference, and Type 2 Diabetes: A Prospective Study

**DOI:** 10.1289/ehp.1307115

**Published:** 2014-05-05

**Authors:** Charlotta Eriksson, Agneta Hilding, Andrei Pyko, Gösta Bluhm, Göran Pershagen, Claes-Göran Östenson

**Affiliations:** 1 Unit of Environmental Epidemiology, Institute of Environmental Medicine, and; 2 Endocrine and Diabetes Unit, Department of Molecular Medicine and Surgery, Karolinska Institutet, Stockholm, Sweden

## Abstract

Background: Long-term aircraft noise exposure may increase the risk of cardiovascular disease, but no study has investigated chronic effects on the metabolic system.

Objectives: The aim of this study was to investigate effects of long-term aircraft noise exposure on body mass index (BMI), waist circumference, and type 2 diabetes. Furthermore, we explored the modifying effects of sleep disturbance.

Methods: This prospective cohort study of residents of Stockholm County, Sweden, followed 5,156 participants with normal baseline oral glucose tolerance tests (OGTT) for up to 10 years. Exposure to aircraft noise was estimated based on residential history. Information on outcomes and confounders was obtained from baseline and follow-up surveys and examinations, and participants who developed prediabetes or type 2 diabetes were identified by self-reported physician diagnosis or OGTT at follow-up. Adjusted associations were assessed by linear, logistic, and random-effects models.

Results: The mean (± SD) increases in BMI and waist circumference during follow-up were 1.09 ± 1.97 kg/m^2^ and 4.39 ± 6.39 cm, respectively. The cumulative incidence of prediabetes and type 2 diabetes was 8% and 3%, respectively. Based on an ordinal noise variable, a 5-dB(A) increase in aircraft noise was associated with a greater increase in waist circumference of 1.51 cm (95% CI: 1.13, 1.89), fully adjusted. This association appeared particularly strong among those who did not change their home address during the study period, which may be a result of lower exposure misclassification. However, no clear associations were found for BMI or type 2 diabetes. Furthermore, sleep disturbances did not appear to modify the associations with aircraft noise.

Conclusions: Long-term aircraft noise exposure may be linked to metabolic outcomes, in particular increased waist circumference.

Citation: Eriksson C, Hilding A, Pyko A, Bluhm G, Pershagen G, Östenson CG. 2014. Long-term aircraft noise exposure and body mass index, waist circumference, and type 2 diabetes: a prospective study. Environ Health Perspect 122:687–694; http://dx.doi.org/10.1289/ehp.1307115

## Introduction

Environmental noise is a stressor, and acute exposure to loud noise has been shown to affect a number of physiological, metabolic, and immunological functions (Babisch 2003; Ising and Kruppa 2004; Spreng 2000a). Noise-induced release of stress hormones, hypothesized to be caused by an increased activity in the sympathetic branch of the autonomic nervous system and hyperactivation of the hypothalamic–pituitary–adrenal axis, is supported by a combination of observational (Babisch et al. 2001; Selander et al. 2009a) and experimental findings (Ising and Braun 2000; Persson Waye et al. 2003). Additionally, long-term exposure to noise has been suggested to cause an imbalance in the stress-regulating mechanism, increasing the risk of cardiovascular diseases [Eriksson et al. 2010; Jarup et al. 2008; Selander et al. 2009b; Sørensen et al. 2011; World Health Organization (WHO) 2011]. Chronically high levels of stress hormones, primarily cortisol, induce hypertonic and diabetogenic effects and may lead to alterations in the adipose tissue metabolism (Björntorp and Rosmond 2000; Pilz and Marz 2008; Spreng 2000b). Compelling evidence also suggests that such a chronic state of stress may contribute to the development of obesity, insulin resistance, and type 2 diabetes (Björntorp 1997; Björntorp and Rosmond 2000; Kyrou and Tsigos 2007; Kyrou et al. 2006; Rosmond 2003, 2005; Rosmond and Björntorp 2000). However, to our knowledge, only one previous study has investigated the link between environmental noise exposure and effects on the metabolic system (Sørensen et al. 2013). This was a large-scale Danish cohort study that reported statistically significant associations between long-term road traffic noise and incidence of diabetes. We are not aware of any previous study of the long-term effects of aircraft noise on the metabolic system.

In addition to evoking a stress response, noise is commonly associated with a disturbed sleep and chronic sleep loss (WHO 2009, 2011). Sleep disturbances affect the general well-being and may have several detrimental health effects, including disruptions of metabolic and endocrine functions (Van Cauter et al. 2008). Sleep debt has been shown to affect the carbohydrate metabolism—for example, reducing glucose tolerance as well increasing the activity of the sympathetic nervous system (Eriksson et al. 2008; Spiegel et al. 1999). Shortened sleep may also affect serum levels of leptin and ghrelin, leading to an increased appetite and reduced energy expenditure, thus increasing the risk of overweight and obesity (Chaput et al. 2007; Taheri et al. 2004). Furthermore, a recent systematic review and meta-analysis on sleep and diabetes showed that both reduced quantity and impaired quality of sleep predicts the risk of developing type 2 diabetes (Cappuccio et al. 2010). However, the role of sleep disturbances as an intermediate factor between aircraft noise exposure and metabolic outcomes remains unexplored.

In two previous publications, we have reported on an association between aircraft noise and cumulative incidence of hypertension among men and women living near Stockholm, Sweden, Arlanda Airport (Eriksson et al. 2007, 2010). In this study, we used the same population to investigate associations between long-term aircraft noise exposure and metabolic outcomes, including body mass index (BMI), waist circumference, and type 2 diabetes. Furthermore, we aimed to assess the modifying effects of several factors, in particular sleep disturbances.

## Methods and Procedures

**Figure 1:**
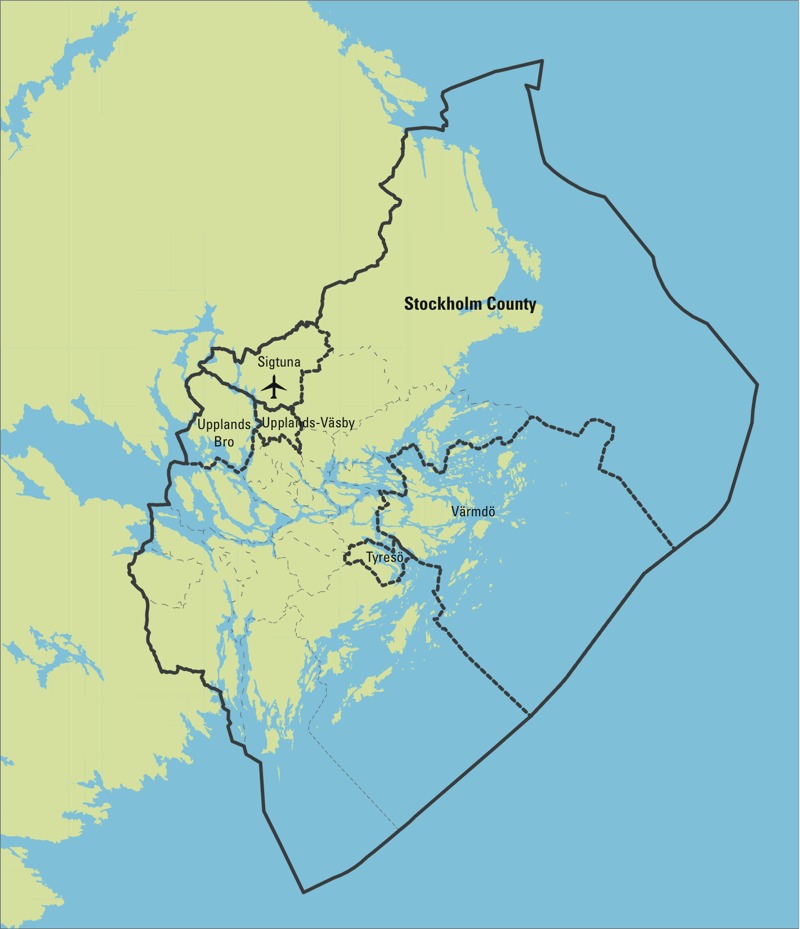
Description of the study area, including five municipalities in Stockholm County: Sigtuna, Upplands Väsby, Upplands Bro, Tyresö, and Värmdö.

*Study population*. This prospective cohort study is based on the Stockholm Diabetes Prevention Program, which was performed between 1992 and 2006 in five municipalities in Stockholm County (Östenson and Bjärås 1995) (Figure 1). The aim of the program was to study risk factors for type 2 diabetes as well as to suggest and implement actions to prevent the disease. Community-based interventions were performed in three of the municipalities: Sigtuna, Upplands Väsby (women only), and Värmdö. Residents of the remaining two municipalities, Upplands Bro and Tyresö, served as reference group. The design of the program has been described in detail previously (Alvarsson et al. 2009; Eriksson et al. 2008, 2010). Briefly, a sample of 3,128 men and 4,821 women 35–56 years of age and without previously diagnosed diabetes were included in a baseline survey between 1992 and 1994 for men and 1996 and 1998 for women. The selection was made so that approximately half of the study participants (52% of the men and 54% of the women) had a family history of diabetes, defined as known diabetes in at least one first-degree relative (mother, father, sister, or brother) or at least two second-degree relatives (grandparents, uncle, or aunt). The other half was a sample without diabetes heredity, frequency matched on age. After 8–10 years, 2002–2004 for men and 2004–2006 for women, all participants of the baseline were invited to a follow-up survey, except those who were diagnosed with type 2 diabetes at baseline, were deceased, or had moved out of Stockholm County during the study period (*n* = 838). Of the remaining 7,111 participants, 2,383 men and 3,329 women took part, corresponding to 76% and 69%, respectively, of the baseline study group. The cohort for analyses was restricted to participants with normal glucose tolerance at baseline (280 persons excluded) and to those with complete exposure and covariate information (21 and 255 persons excluded, respectively), resulting in a study population of 5,156 participants.

The study was approved by the Karolinska Hospital Research Ethics Committee, and all participants gave their informed consent.

**Figure 2:**
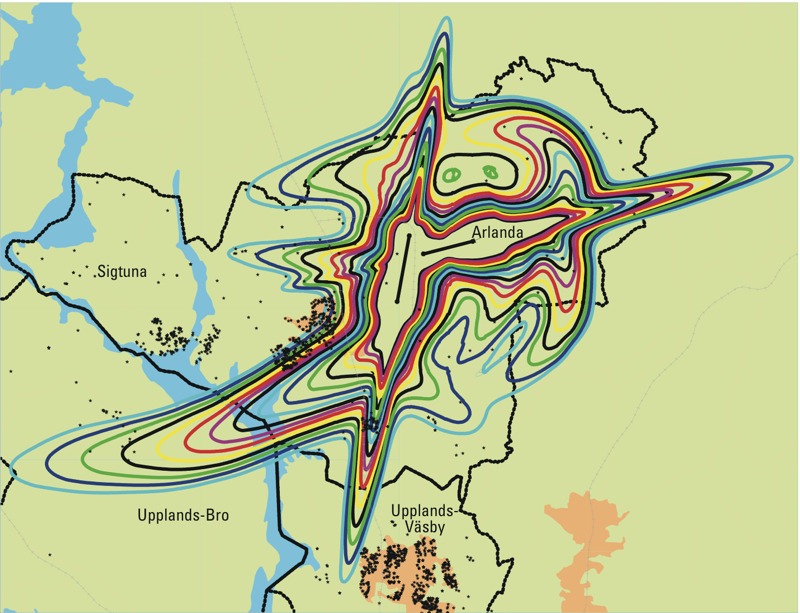
Aircraft noise exposure [represented by colors; noise levels range from 50 (outer line) to 65 dB (inner line)] and study participants’ addresses (black stars) around Stockholm Arlanda Airport.

*Exposure assessment*. The method for estimating aircraft noise exposure has been described previously (Eriksson et al. 2010). In summary, the exposure assessment was made using geographic information systems, and is based on residential histories of the participants. The addresses, obtained from the Swedish Population Register (Swedish Tax Agency, Solna, Sweden) and through questionnaire answers, were geocoded by the Swedish Mapping, Cadastre and Land Registration Authority (Gävle, Sweden) and plotted on a digital map of Stockholm County together with 1-dB resolution contours of the aircraft noise exposure around Stockholm Arlanda Airport, located in the municipality of Sigtuna (Figure 2). Aircraft noise exposure ranging from 50 to 65 dB(A) *L*_den_ was modeled by the Swedish Airports and Air Navigation Services, using the Integrated Noise Model, version 6.1 [ECAC (European Civil Aviation Conference)-CEAC (Conférence Européenne de l’Aviation Civile) 2005]. *L*_den_ is the A-weighted 24-hr equivalent continuous sound pressure level, with an addition of 5 dB for evening noise events (in Sweden, defined as the period 1900–2300 hours) and 10 dB for night time noise events (in Sweden, 2300–0700 hours) (European Commission 2002).

As a consequence of the introduction of new quieter aircrafts, the exposure around Arlanda decreased continuously during the study period. To account for this decline, and because detailed aircraft noise data were not obtainable until a radar tracking system was introduced at the airport in the early 21st century, we used the average aircraft noise level for 1997–2002 as an indicator of noise exposure for the complete study period. The exposure was estimated from radar tracks for the year 2002 and corrected for the prevailing traffic situation during the preceding 5-year period. Some changes in the exposure took place in 2003 when a third runway was taken into operation. This affected primarily the municipality of Upplands Väsby, where only women were included (845 of the total of 3,065 women). However, these alterations have not been considered because they occurred late in our study period and affected only a smaller proportion of our participants.

Approximately 27% of the participants moved residence during the study period, and for these, we calculated a time-weighted mean value of exposure. Participants who were exposed to aircraft noise during only part of the follow-up period were assumed to have been exposed to a background noise level of 47 dB(A) during the time they lived at unexposed addresses. This level was based on municipality mappings of road traffic in or study area.

**Figure 3:**
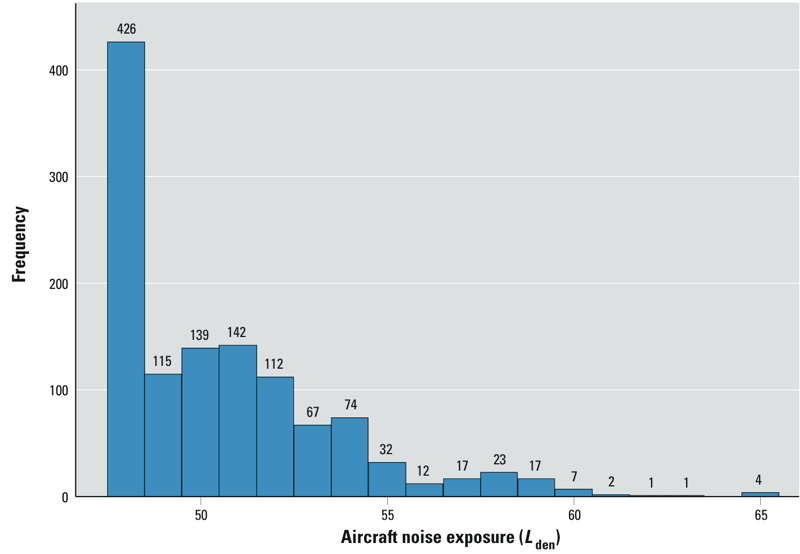
Exposure distribution among 1,191 aircraft noise–exposed participants (unexposed participants not included, *n* = 3,965). Numbers above bars indicate frequencies.

Among our study participants, 650 (13%) were exposed to average aircraft noise levels ≥ 50 dB(A) *L*_den_ (Figure 3). Additionally, 541 (11%) had been partially exposed to aircraft noise during the study period and had an estimated time-weighted average exposure of 48 or 49 dB(A) *L*_den_.

*Assessment and definitions of outcomes*. The baseline and follow-up surveys included extensive questionnaires as well as clinical examinations and were carried out at primary health care centers, always during the mornings and with participants fasting overnight. The questionnaires asked about general health and lifestyle, including dietary habits, physical activity, and tobacco use, symptoms or medication, education, occupation, and social contacts. At follow-up, questions regarding noise annoyance were also included. The health examinations were performed by trained nurses and included measurements of blood pressure, weight, and height as well as waist and hip circumference. For each individual, BMI was calculated as the weight divided by the squared height (kilograms per meter squared). The examination also included an oral glucose tolerance test (OGTT), in which levels of plasma/serum glucose (millimoles per liter) were measured before (i.e., fasting glucose) and 2 hr after glucose ingestion. Based on the results, the participants were classified in groups of normal glucose tolerance (NGT), impaired fasting glucose (IFG), impaired glucose tolerance (IGT), or manifest type 2 diabetes, according to WHO standards (WHO 1999).

Participants with an IFG and/or IGT at the follow-up examination were defined as having prediabetes. Furthermore, those who were classified as having manifest type 2 diabetes at follow-up or reported being diagnosed with type 2 diabetes by a physician during the study period were defined as having type 2 diabetes.

*Statistical analyses*. Associations between aircraft noise and changes in BMI and waist circumference from baseline to follow-up were estimated using linear and random-effects regression models to derive regression coefficients (β) and 95% CIs. Because 45 participants had missing data on BMI and/or waist circumference, the analyses were restricted to those with complete data on these outcomes (*n* = 5,111). Both outcomes were normally distributed (data not shown). Associations between aircraft noise and cumulative incidence of prediabetes, type 2 diabetes, and prediabetes or type 2 diabetes (combined) were analyzed using logistic and random-effects regression models to estimate odds ratios (OR) and 95% CIs.

Aircraft noise was included in the models both as a binary variable, estimating associations with aircraft noise ≥ 50 versus < 50 dB(A) (including participants who were unexposed), and as ordinal variables grouped in three and six categories, respectively. The variable grouped in three categories were coded as 0 for unexposed participants and for those with an exposure < 50, 1 if 50–54 and 2 if ≥ 55 dB(A). The categorization was used to evaluate an exposure-response pattern by comparing group 1 and 2 to the reference group, coded as 0. For the variable grouped in six categories we used the following coding: 0 if unexposed, 1 if the exposure was < 50, 2 if 50–54, 3 if 55–59, 4 if 60–64, and 5 if ≥ 65 dB(A) and estimates are given per unit increase of this variable, approximate to 5 dB(A).

Individual-level variables included as final model covariates were selected using a backward variable selection technique (*p* for inclusion < 0.05). All variables except noise annoyance were classified according to baseline values. The variables which were assessed included sex, age (35–39, 40–44, 45–49, 50–55 years), family history of diabetes (negative or positive), socioeconomic status based on occupation (manual workers, low-level non-manual workers, medium- and high-level non-manual workers, and self-employed and farmers), physical activity [low (sedentary lifestyle), moderate (occasional exercise), or high (regular exercise or training)], tobacco use (never, former, or current smoking or use of moist snuff), alcohol consumption (low, medium, or high) and annoyance due to noise from other sources, including road, rail, or occupational noise [not annoyed (seldom/never or annoyed a few times per month) or annoyed (annoyed a few times per week or every day)]. Quality of diet was assessed by recommended and nonrecommended food scores (Kaluza et al. 2009; Michels and Wolk 2002). Among the recommended foods we included consumption of low-fat dairy products, whole-meal or hard bread, fruits, vegetables (score + 1 if consumed at least 2–3 times per week), and porridge (+ 1 if consumed at least 1–3 times per month). Among the nonrecommended foods we included consumption of high-fat dairy products, white bread (score + 1 if consumed at least 2–3 times per week), fast foods (+ 1 if consumed at least 1–3 times per month), cakes and sweets (+ 1 if consumed at least once a week). Summarized, the two scores for recommended and nonrecommended foods ranged from 0 to 14 and 0 to 12, respectively. The scores were then categorized in quartiles and combined into a five-category total food score, ranging from poor (low on recommended foods and high on nonrecommended) to excellent (high on recommended and low on nonrecommended). We also assessed job strain, which was based on the Swedish version of the Karasek and Theorell demand-decision latitude questionnaire (Agardh et al. 2003; Karasek 1979). From the questions, two indices for work-related demands and decision latitude were created that were further categorized in tertiles. Job strain was defined as the highest tertile of demand together with the lowest tertile of decision latitude. A similar index was created for psychological distress which was assessed from questions on anxiety, apathy, depression, fatigue, and insomnia (Eriksson et al. 2008). Sleep disturbance [classified as not disturbed (never or seldom) or disturbed (sometimes or often)] was considered as a possible intermediate factor in the causal pathway and therefore was not included as a confounder. All baseline individual-level variables that were statistically significant (*p* < 0.05) predictors of any of the outcomes were included in the final individual-level models and included sex, age, family history of diabetes, socioeconomic status, physical activity, tobacco use, and psychological distress.

In fully adjusted models, we also adjusted for contextual confounding by area-level mean income (yearly) and proportion of unemployed (%), using data from Statistics Sweden (http://www.scb.se/en_/). These variables were classified according to residence at baseline. The analyses were performed using linear and logistic random-effects models, clustering on 139 small areas considered homogenous with respect to socioeconomic characteristics.

Differences in background and follow-up characteristics according to level of exposure were assessed by Pearson chi-square tests for categorical variables and one-way analysis of variance (ANOVA) for continuous variables. The default α level was set at 0.05.

All of the above-mentioned individual-level covariates and additionally noise annoyance (not annoyed, i.e., never/seldom or a few times per month; or annoyed, i.e., a few times per week or every day) and changing home address during the study period (yes/no) were investigated with regard to effect modification. However, due to low power, the analyses for diabetes were performed only for sex, age, family history of diabetes, physical activity, sleep disturbances, aircraft noise annoyance, and changing home address. The covariates were included in the models as interaction terms with the binary exposure variable [< 50 vs. ≥ 50 dB(A)], using a Wald test to assess statistical significance of overall interaction (α level for effect modification = 0.10).

## Results

The mean follow-up time in our population was 8.9 years. The mean increase in BMI between baseline and follow-up was 1.09 kg/m^2^ ± 1.97, and mean increase in waist circumference was 4.39 cm ± 6.39. In total, we identified 412 cases of prediabetes and 159 cases of type 2 diabetes during the study period, corresponding to cumulative incidences of 8% and 3% (of 5,516 participants), respectively.

Significant differences in baseline characteristics among the noise exposure groups were found for sex, family history of diabetes, socioeconomic status, and physical activity, as well as for mean income and unemployment on the area level (Table 1). Furthermore, at follow-up, there were significant differences in aircraft noise annoyance, annoyance due to noise from other sources, changes in BMI and waist circumference, as well as type 2 diabetes (Table 2).

**Table 1:** Baseline characteristics of participants in the Stockholm Diabetes Prevention Program according to aircraft noise level^*a*^ (*n* = 5,156).

Baseline characteristics^*b*^	< 50 dB	50–54 dB	≥ 55 dB	*p*-Value^*c*^
Total	4,506 (100)	534 (100)	116 (100)
Sex (male)	1,783 (40)	252 (47)	56 (48)	0.001
Age (years)	47 ± 4.90	47 ± 5.20	47 ± 5.06	0.623
Family history of diabetes	2,310 (51)	299 (56)	71 (61)	0.016
Socioeconomic status				< 0.001
Manual workers	1,149 (26)	193 (36)	39 (34)
Low-level non-manual workers	1,006 (22)	122 (23)	22 (19)
Medium- and high-level non-manual	2,145 (48)	208 (39)	50 (43)
Self-employed and farmers	206 (5)	11 (2)	5 (4)
Physical activity				0.016
Low	431 (10)	60 (11)	16 (14)
Moderate	2,404 (53)	313 (59)	59 (51)
High	1,671 (37)	161 (30)	41 (35)
Tobacco use				0.174
Never	1,670 (37)	181 (34)	46 (40
Former	1,490 (33)	167 (31)	34 (29)
Current	1,346 (30)	186 (35)	36 (31)
Alcohol consumption				0.058
Low	1,454 (33)	205 (39)	33 (28)
Medium	1,516 (34)	174 (33)	43 (37)
High	1,472 (33)	153 (29)	40 (34)
Total Food Score^*d*^				0.565
Poor	1,003 (22)	120 (22)	35 (30)
Inadequate	805 (18)	103 (19)	19 (16)
Fair	1,062 (24)	131 (25)	24 (21)
Good	825 (18)	98 (18)	20 (17)
Excellent	811 (18)	82 (15)	18 (16)
Job strain	365 (8)	55 (11)	13 (12)	0.113
Psychological distress^*e*^				0.633
Quartile 1	1,054 (23)	120 (22)	27 (23)
Quartile 2	1,135 (25)	124 (23)	36 (31)
Quartile 3	414 (31)	179 (34)	31 (27)
Quartile 4	903 (20)	111 (21)	22 (19)
Sleep disturbances	263 (28)	157 (29)	27 (23)	0.408
Mean income on area level (SEK)	296,223 ± 47,820	246,311 ± 41,533	278,994 ± 32,694	< 0.001
Unemployment on area level (%)	2.32 ± 1.24	3.15 ± 1.87	2.29 ± 1.21	< 0.001
Values are *n* (%) or mean ± SD. ^***a***^*L*_den_: the A-weighted 24-hr equivalent continuous sound pressure level, with an addition of 5 dB for evening noise events (in Sweden, defined as the period 1900–2300 hours) and 10 dB for nighttime noise events (in Sweden, 2300–0700 hours). ^***b***^Less than 5% missing for each variable. ^***c***^*p*-Values were assessed by Pearson chi-square tests for categorical variables and one-way ANOVA for continuous variables. Alpha = 0.05. ^***d***^Food Score Index based on consumption of recommended and non-recommended food items. ^***e***^Quartiles of an index created from questions on anxiety, apathy, depression, fatigue, and insomnia.				

**Table 2:** Follow-up characteristics of participants in the Stockholm Diabetes Prevention Program according to aircraft noise level^*a*^ (*n* = 5,156).

Follow-up characteristics^*b*^	< 50 dB	50–54 dB	≥ 55 dB	*p*-Value^*c*^
Total
All participants	4,506 (100)	534 (100)	116 (100)
Men	1,783 (100)	252 (100)	56 (100)
Women	2,723 (100)	282 (100)	60 (100)
Annoyance, aircraft noise
All participants	414 (9)	142 (27)	67 (58)	< 0.001
Men	95 (5)	76 (30)	34 (61)	< 0.001
Women	319 (11)	66 (23)	33 (55)	< 0.001
Annoyance, other noise sources
All participants	1,099 (24)	154 (29)	39 (34)	< 0.001
Men	338 (19)	65 (26)	18 (32)	< 0.001
Women	761 (28)	89 (32)	21 (35)	0.548
BMI difference^*d*^
All participants	1.06 ± 1.97	1.29 ± 2.04	1.12 ± 1.78	0.04
Men	1.18 ± 1.79	1.42 ± 1.72	1.05 ± 1.79	0.113
Women	0.98 ± 2.07	1.16 ± 2.29	1.18 ± 1.78	0.288
Waist difference^*e*^
All participants	3.95 ± 6.31	7.49 ± 6.01	7.23 ± 6.37	< 0.001
Men	2.73 ± 6.37	6.66 ± 5.64	7.02 ± 6.86	< 0.001
Women	4.75 ± 6.20	8.23 ± 6.23	7.43 ± 5.92	< 0.001
Prediabetes^*f*^
All participants	360 (8)	40 (7)	12 (10)	0.590
Men	205 (12)	24 (10)	7 (13)	0.624
Women	155 (6)	16 (6)	5 (8)	0.684
Type 2 diabetes^*g*^
All participants	133 (3)	22 (4)	4 (3)	0.327
Men	88 (5)	13 (5)	1 (2)	0.546
Women	45 (2)	9 (3)	3 (5)	0.036
Pre- and type 2 diabetes
All participants	493 (11)	62 (12)	16 (14)	0.575
Men	293 (16)	37 (15)	8 (14)	0.723
Women	200 (7)	25 (9)	8 (13)	0.157
Values are *n* (%) or mean ± SD. ^***a***^*L*_den_: The A-weighted 24-hr equivalent continuous sound pressure level, with an addition of 5 dB for evening noise events (in Sweden defined as the period 1900–2300 hours) and 10 dB for night time noise events (in Sweden: 2300–0700 hours). ^***b***^Less than 1% missing for each variable. ^***c***^*p*-Values were assessed by Pearson chi-square tests for categorical variables and one-way analysis of variance for continuous variables; α = 0.05. ^***d***^Difference in BMI (kg/m^2^) from baseline to follow-up. ^***e***^Difference in waist circumference (cm) from baseline to follow-up. ^***f***^Impaired fasting glucose and/or glucose tolerance at follow-up. ^***g***^Physician diagnosis during the study period or identified via the oral glucose tolerance test at follow-up.				

After adjustments for individual-level confounders, long-term aircraft noise exposure was associated with a 0.08-kg/m^2^ (95% CI: 0.01, 0.15) increase in BMI between baseline and follow-up per unit increase in the ordinal noise variable grouped in six categories (data not shown). However, in the fully adjusted model, no association was found (Table 3).

**Table 3:** Associations [β (95% CI)] between aircraft noise exposure and changes in BMI (kg/m^2^) and waist circumference (cm) from baseline to follow-up (*n* = 5,111).

Aircraft noise exposure (*L*_den_)^*a*^	BMI^*b*^	Waist circumference^*b*^
Dichotomous [dB(A)]
All participants
< 50	0 (reference)	0 (reference)
≥ 50	0.05 (–0.15, 0.25)	1.34 (0.52, 2.16)
Men
< 50	0 (reference)	0 (reference)
≥ 50	0.11 (–0.12, 0.34)	1.93 (0.85, 3.00)
Women
< 50	0 (reference)	0 (reference)
≥ 50	0.05 (–0.19, 0.30)	2.26 (1.23, 3.30)
Ordinal, three categories [dB(A)]
All participants
< 50	0 (reference)	0 (reference)
50–54	0.08 (–1.14, 0.29)	1.31 (0.45, 2.16)
≥ 55	–0.08 (–0.49, 0.32)	1.51 (–0.05, 3.07)
Men
< 50	0 (reference)	0 (reference)
50–54	0.16 (–0.08, 0.41)	1.80 (0.67, 2.94)
≥ 55	–0.12 (–0.59, 0.35)	2.41 (0.29, 4.52)
Women
< 50	0 (reference)	0 (reference)
50–54	0.04 (–0.23, 0.31)	2.29 (1.20, 3.37)
≥ 55	0.11 (–0.42, 0.64)	2.09 (0.11, 4.07)
Ordinal, six categories^*c*^
All participants	0.04 (–0.05, 0.13)	1.51 (1.13, 1.89)
Men	0.07 (–0.03, 0.16)	2.26 (1.83, 2.69)
Women	0.05 (–0.05, 0.15)	1.58 (1.13, 2.03)
^***a***^*L*_den_: the A-weighted 24-hr equivalent continuous sound pressure level, with an addition of 5 dB for evening noise events (in Sweden, defined as the period 1900–2300 hours) and 10 dB for nighttime noise events (in Sweden, 2300–0700 hours). ^***b***^Random-effects linear regression model adjusted for sex, age, family history of diabetes, socioeconomic status, physical activity, tobacco use and psychological distress on individual level as well as mean income (yearly) and ­unemployment (%) on area level. ^***c***^Increment per unit increase, approximate to 5 dB(A) *L*_den_.		

Waist circumference was clearly associated with aircraft noise in all models, showing a statistically significant increase of 2.14 cm (95% CI: 1.93, 2.35) per unit increase in the six-category ordinal noise variable when adjusting for individual-level confounders only (data not shown). The association was closer to the null but remained statistically significant in the fully adjusted model, with an estimated increase of 1.51 cm (95% CI: 1.13, 1.89) per unit increase in the six-category ordinal noise variable. Furthermore, a monotonic exposure–response pattern was evident in the population as a whole, with a regression coefficient of 1.31 cm (95% CI: 0.45, 2.16) among those exposed at *L*_den_ 50–54 dB(A) and 1.51 cm (95% CI: –0.05, 3.07) among those exposed at ≥ 55 dB(A), though the pattern was not monotonic for women.

There were no significant associations between aircraft noise and cumulative incidence of prediabetes and/or type 2 diabetes in the overall population; the fully adjusted OR for the two outcomes combined was 0.93 (95% CI: 0.82, 1.06) per unit increase in the six-category ordinal noise variable (Table 4). For type 2 diabetes, a monotonic exposure–response pattern was present for women, with an OR of 1.51 (95% CI: 0.69, 3.32) among those exposed at *L*_den_ 50–54 dB(A) and 2.78 (95% CI: 0.80, 9.60) among those exposed at ≥ 55 dB(A), but not for men (OR = 0.83; 95% CI: 0.42, 1.63 and 0.31; 95% CI: 0.04, 2.38, respectively). However, the estimates for the highest exposure group were based on only three and one exposed cases for women and men, respectively.

**Table 4:** Associations [OR (95% CI)] between aircraft noise exposure and cumulative incidence of prediabetes (*n* = 412 cases), type 2 diabetes (*n* = 159 cases), and prediabetes and type 2 diabetes combined (total *n* = 5,156).

Aircraft noise exposure (*L*_den_)^*a*^	Total *n*	Prediabetes^*b*^		Type 2 diabetes^*c*^		Combined	
		*n* (%)	OR (95% CI)^*d*^	*n* (%)	OR (95% CI)^*d*^	*n* (%)	OR (95% CI)^*d*^
Dichotomous [dB(A)]
All participants
< 50	4,506	360 (8)	1.00 (reference)	133 (3)	1.00 (reference)	493 (11)	1.00 (reference)
≥ 50	650	52 (8)	0.86 (0.62, 1.21)	26 (4)	0.98 (0.61, 1.59)	78 (12)	0.87 (0.64, 1.18)
Men
< 50	1,783	205 (12)	1.00 (reference)	88 (5)	1.00 (reference)	293 (16)	1.00 (reference)
≥ 50	308	31 (10)	0.89 (0.58, 1.37)	14 (5)	0.73 (0.38, 1.40)	45 (15)	0.81 (0.54, 1.20)
Women
< 50	2,723	155 (6)	1.00 (reference)	45 (2)	1.00 (reference)	200 (7)	1.00 (reference)
≥ 50	342	21 (6)	0.86 (0.53, 1.41)	12 (4)	1.73 (0.86, 3.48)	33 (10)	1.07 (0.71, 1.61)
Ordinal, three categories [dB(A)]
All participants
< 50	4,506	360 (8)	1.00 (reference)	133 (3)	1.00 (reference)	493 (11)	1.00 (reference)
50–54	534	40 (7)	0.80 (0.55, 1.16)	22 (4)	1.00 (0.60, 1.66)	62 (12)	0.83 (0.60, 1.16)
≥ 55	116	12 (10)	1.15 (0.61, 2.19)	4 (3)	0.94 (0.33, 2.70)	16 (14)	1.06 (0.58, 1.94)
Men
< 50	1,783	205 (12)	1.00 (reference)	88 (5)	1.00 (reference)	293 (16)	1.00 (reference)
50–54	252	24 (10)	0.84 (0.52, 1.36)	13 (5)	0.83 (0.42, 1.63)	37 (15)	0.82 (0.53, 1.25)
≥ 55	56	7 (13)	1.08 (0.47, 2.51)	1 (2)	0.31 (0.04, 2.38)	8 (14)	0.77 (0.34, 1.76)
Women
< 50	2,723	155 (6)	1.00 (reference)	45 (2)	1.00 (reference)	200 (7)	1.00 (reference)
50–54	282	16 (6)	0.79 (0.45, 1.37)	9 (3)	1.51 (0.69, 3.32)	25 (9)	0.95 (0.60, 1.51)
≥ 55	60	5 (8)	1.23 (0.47, 3.17)	3 (5)	2.78 (0.80, 9.60)	8 (13)	1.62 (0.74, 3.54)
Ordinal, six categories^*e*^
All participants	5,156	412 (8)	0.91 (0.78, 1.04)	159 (3)	1.03 (0.84, 1.26)	571 (11)	0.93 (0.82, 1.06)
Men	2,091	236 (11)	0.92 (0.76, 1.11)	102 (5)	0.92 (0.70, 1.21)	338 (16)	0.91 (0.77, 1.08)
Women	3,065	176 (6)	0.89 (0.72, 1.11)	57 (4)	1.27 (0.94, 1.71)	233 (8)	0.99 (0.83, 1.19)
^***a***^*L*_den_: the A-weighted 24-hr equivalent continuous sound pressure level, with an addition of 5 dB for evening noise events (in Sweden, defined as the period 1900–2300 hours) and 10 dB for nighttime noise events (in Sweden, 2300–0700 hours). ^***b***^Impaired fasting glucose and/or glucose tolerance. ^***c***^Physician diagnosis during the study period or identified at follow-up. ^***d***^Random-effects logistic regression model adjusted for sex, age, family history of diabetes, socioeconomic status, physical activity, tobacco use, and psychological distress on individual level as well as mean income (yearly) and unemployment (%) on area level. ^***e***^Increment per unit increase, approximate to 5 dB(A) *L*_den_.							

The analyses of effect modification showed few consistent results, and, for prediabetes and type 2 diabetes, had to be restricted to covariates where we had a sufficient number of exposed cases. For participants who had high job strain, the association of ≥ 50 versus < 50 dB(A) with change in BMI was 0.48 kg/m^2^ (95% CI: –0.04, 0.99) compared with 0.02 kg/m^2^ (95% CI: –0.19, 0.24; *p*_interaction_ = 0.094) among those with low job strain, and the association with change in waist circumference was 2.40 cm (95% CI: 0.74, 4.07) compared with 1.08 cm (95% CI: 0.22, 1.94; *p*_interaction_ = 0.108), respectively (see Supplemental Material, Table S1). For participants who did not change their home address, the association of ≥ 50 versus < 50 dB(A) with change in waist circumference was 2.64 cm (95% CI: 0.96, 4.31) compared with 1.69 cm (95% CI: 0.80, 2.58; *p*_interaction_ = 0.096) among participants who moved during the study period. The association of prediabetes with noise ≥ 50 versus < 50 dB(A) was significantly lower among those with high physical activity (OR = 0.37; 95% CI: 0.16, 0.87) compared with low physical activity (OR = 1.05; 95% CI: 0.73, 1.51; *p*_interaction_ = 0.024) (see Supplemental Material, Table S2). For participants who did not change their home address, the OR of ≥ 50 versus < 50 dB(A) with prediabetes was 2.17 (95% CI: 0.78, 6.02) compared with 1.01 (95% CI: 0.70, 1.46; *p*_interaction_ = 0.070) among those moving. For type 2 diabetes, there was also a statistically significant effect modification by sex, with ORs associated with noise ≥ 50 versus < 50 dB(A) of 0.70 (95% CI: 0.38, 1.30) for males and 1.68 (95% CI: 0.85, 3.31; *p*_interaction_ = 0.053) for females. Sleep disturbances were not related to any of the outcomes and did not appear to modify the effects of aircraft noise exposure.

## Discussion

To our knowledge, this is the first study of long-term aircraft noise exposure and metabolic outcomes. The main finding of our study was an association between aircraft noise exposure and increased waist circumference, which was statistically significant and showed a monotonic exposure–response pattern after adjustment for individual- and area-level confounders. No clear associations were found for BMI or prediabetes, and although there was a monotonic exposure–response pattern for type 2 diabetes among women, the findings did not reach statistical significance and were not consistent for men.

Although there is a lack of epidemiologic studies linking long-term noise exposure to overweight or obesity, substantial evidence links noise to a stress response (Babisch 2003; Babisch et al. 2001; Ising and Braun 2000; Ising and Kruppa 2004; Persson Waye et al. 2003; Selander et al. 2009a; Spreng 2000a), and also links chronic stress to impaired metabolic functions (Björntorp and Rosmond 2000; Kyrou and Tsigos 2007; Rosmond 2003, 2005; Rosmond and Björntorp 2000; Spreng 2000b). In addition, noise exposure is commonly associated with sleep disturbances, which are known to have metabolic complications (Cappuccio et al. 2010; Chaput et al. 2007; Spiegel et al. 1999; Taheri et al. 2004; Van Cauter et al. 2008). As mentioned, we found an association between aircraft noise and increases in waist circumference; however, the findings for BMI were not as clear. Possible explanations may include a development of central rather than generalized obesity caused by a noise-induced cortisol secretion (Björntorp 1997; Kyrou et al. 2006). Chronic stress is characterized by hyperactivation of the hypothalamic–pituitary–adrenal axis and may impair the feedback control of central glucocorticoid receptors. Elevated levels of cortisol lead to storage of fat in visceral depots, especially in the abdominal area. However, future studies are needed to confirm this potential link between noise, stress, and central obesity.

No clear associations were found between aircraft noise and prediabetes or type 2 diabetes in the overall population. However, for type 2 diabetes, effect modification by sex was indicated, with stronger associations among women. There is some evidence from previous literature of a stronger association between noise and metabolic markers in women. For example, the cross-sectional HYENA (Hypertension and Exposure to Noise near Airports) study estimated associations of aircraft noise exposure with saliva cortisol in 439 men and women living near major airports in six European countries (Selander et al. 2009a). On average, women exposed to noise levels > 60 dB *L*_Aeq,24h_ had significantly higher morning saliva cortisol concentrations than women exposed to < 50 dB (β = 6.07 mmol/L; 95% CI: 2.32, 9.81), consistent with a noise-induced stress reaction. No such association was seen for men (β = –2.00 mmol/L; 95% CI: –5.61, 1.61). Furthermore, a recent population-based cohort study among 57,053 Danish residents reported an association between road traffic noise and diabetes (Sørensen et al. 2013). In this study, the incidence rate ratio for a 10-dB(A) *L*_den_ increase in average noise exposure during the 5 years preceding diagnosis was 1.11 (95% CI: 1.05, 1.18). Also, associations were stronger among females than among males. Yet, additional large-scale longitudinal studies are needed to clarify sex-specific associations between noise and metabolic outcomes.

Some of the individual characteristics we examined significantly modified associations with aircraft noise. High job strain, which was previously reported to be a possible effect modifier of the association between road traffic noise and myocardial infarction (Selander et al. 2013), was associated with greater increases in both BMI and waist circumference among participants exposed to aircraft noise levels ≥ 50 dB(A) compared with those exposed below this level. Thus, multiple stressors may add to the individual’s stress load in a negative way. On the other hand, the association between noise and prediabetes was decreased among those with high physical activity compared with those with low activity, suggesting a buffering effect on the stress load. Furthermore, not changing home address during the study period was associated with stronger associations between aircraft noise and prediabetes as well as waist circumference, possibly a result of reduced exposure misclassification in this group. Unfortunately, small numbers of exposed cases of prediabetes and type 2 diabetes prohibited more detailed analyses of effect modification for these outcomes.

Sleep loss may have metabolic consequences by interfering with glucose regulation, control of appetite, and energy expenditure (Eriksson et al. 2008; Taheri et al. 2004; Van Cauter et al. 2008). However, in this study sleep disturbances were related neither to aircraft noise, possibly due to insulation of the most highly exposed residences, nor to any of the outcomes. Furthermore, our analyses of effect modification did not support the hypothesis of a moderating role of sleep disturbances on the association between aircraft noise and metabolic outcomes. However, an effect of sleep on metabolic outcomes should not be excluded because our assessment of sleep disturbances was based on self-report and rather crude.

Area-level socioeconomic factors may constitute strong sources of confounding in studies on environmental factors and health (Chaix et al. 2010). Because our study area included five different municipalities in Stockholm County—three in the northwest, close to Stockholm Arlanda airport, and an additional two in the southeast—we were concerned that regional differences in socioeconomic status might influence our results. In addition to individual-level factors, we therefore made adjustments for area-level mean income (yearly) and the proportion of unemployed residents. Neither mean income nor the proportion of unemployed was highly correlated to individual socioeconomic status, and adjustments for these factors tended to reduce the risk estimates for aircraft noise. This suggests that the association between aircraft noise and the outcomes may have been influenced by regional differences in socioeconomic status.

A limitation of our study is the narrow range of exposure and the small number of highly exposed cases. This was particularly evident for type 2 diabetes, where we had only 47 cases who had ever been exposed to aircraft noise and only 26 cases exposed at ≥ 50 dB(A). Thus, the associations between aircraft noise and prediabetes and type 2 diabetes in our study are uncertain.

Another limitation is the lack of objective data on exposure to noise from other sources, such as road traffic, railways, and occupation. Such sources may cause confounding, and though we adjusted for annoyance from these sources, some residual confounding may be present, particularly from road traffic and railway noise. Also, as described in a previous publication (Eriksson et al. 2010), the exposure to aircraft noise may have been underestimated for males because men were followed from an earlier time point, when the noise exposure was higher, than women were. Furthermore, women in Upplands Väsby may have been misclassified with regard to exposure because the opening of the third runway in 2003 led to increased aircraft traffic in this area. In fact, one-third of these women reported being disturbed by aircraft noise, although few of them were classified as exposed according to our exposure estimate. However, because we studied outcomes that develop during an extended period of time, changes in the noise exposure occurring late in the study period would not be expected to be of major importance.

Because only one OGTT was performed, there is some uncertainty in the classification of prediabetes and type 2 diabetes. The reproducibility of an OGTT may be reduced due to variation in the quality of the glucose measurements as well as intraindividual variations. In a systematic review of five studies, the reproducibility of a single test was 33–45% for IGT, 51–64% for IFG, and 73% for diabetes (Balion et al. 2007). Another reason for low reproducibility and misclassification is regression to the mean (Yudkin and Stratton 1996), indicating that individuals selected because they have a single high measurement will include a disproportionate number of individuals whose measurement by chance was higher than its true value. In our study, this may have led to misclassification of glucose tolerance.

Furthermore, our cohort oversampled persons with a family history of diabetes (approximately 50%, compared with 20–25% in the general population). Although we did not detect any statistically significant difference in the effects of noise exposure among those with family history of diabetes compared with those with no such history, the associations between aircraft noise and BMI as well as waist circumference appeared stronger among those without family history of diabetes. This could influence the possibilities to generalize our finding to the population as a whole.

Finally, the strengths of this study include its longitudinal design and the objective and independent estimates of the exposure as well as the outcomes. Information from questionnaires, clinical examinations, and high-quality registers (for area-level data) also enabled adjustment for potentially important individual and contextual confounders. Nevertheless, residual confounding may be present.

## Conclusions

In conclusion, we estimated a statistically significant positive association between long-term aircraft noise exposure and change in waist circumference over time. These findings provide evidence of a link between aircraft noise and metabolic outcomes, especially central obesity. However, additional large-scale longitudinal studies are needed to confirm the association.

## Correction

In Table 2 of the manuscript originally published online, the waist-difference values for women were incorrect. They have been corrected here.

## Supplemental Material

(188 KB) PDF
