# Correction: Long-Term Aircraft Noise Exposure and Body Mass Index, Waist Circumference, and Type 2 Diabetes: A Prospective Study

**DOI:** 10.1289/ehp.1307115-erratum

**Published:** 2014-05-05

In Table 2 of the manuscript originally published online, the waist-difference values for women were incorrect. They have been corrected here.

