# Supplementary material for: Long-Term Aircraft Noise Exposure and Body Mass Index, Waist Circumference, and Type 2 Diabetes: A Prospective Study
Source: Environ Health Perspect. 2014 May 5;122(7):687–94. doi: 10.1289/ehp.1307115 (PMC4080526; doi:10.1289/ehp.1307115)
Supplement: (188 KB) PDF [file ehp.1307115.s001.pdf]

Environmental Health Perspectives doi:10.1289/ehp.1307115

Supplemental Material

Long-Term Aircraft Noise Exposure and Body Mass Index, Waist Circumference, and Type 2 Diabetes: A Prospective Study

Charlotta Eriksson, Agneta Hilding, Andrei Pyko, Gösta Bluhm, Göran Pershagen, and Claes-Göran Östenson

Table S1. Associations [β (95% CI)] of aircraft noise [≥50 versus <50)] with change in body mass index (BMI kg/m^2) and waist circumference (cm) from baseline to follow-up according to potential effect modifiers (N=5,111).

Predictors[a]	N	BMI[b]	p-value[c]	Waist circumference[b]	p-value[c]
**Sex**			0.994		0.659
Men	2,069	0.08 (-0.16, 0.33)		1.54 (0.57, 2.51)	
Women	3,042	0.08 (-0.14, 0.31)		1.31 (0.39, 2.25)	
**Age**			0.924		0.158
35-39	525	0.17 (-0.33, 0.67)		0.96 (-0.65, 2.58)	
40-44	1,021	0.10 (-0.27, 0.47)		0.31 (-0.95, 1.57)	
45-49	1,724	-0.01 (-0.31, 0.29)		1.51 (0.43, 2.58)	
50-55	1,841	0.03 (-0.27, 0.33)		1.84 (0.77, 2.90)	
**Family history of diabetes**			0.130		0.244
Negative	2,460	0.18 (-0.12, 0.47)		1.48 (0.48, 2.49)	
Positive	2,651	-0.08 (-0.35, 0.19)		0.89 (-0.07, 1.85)	
**Socioeconomic status**			0.791		0.621
Manual workers	1,367	0.02 (-0.26, 0.30)		1.64 (0.58, 2.69)	
Low-level non-manuals	1,142	0.04 (-0.30, 0.39)		0.83 (-0.39, 2.05)	
Medium and high level non-manuals	2,382	0.12 (-0.14, 0.38)		1.53 (0.50, 2.55)	
Self-employed and farmers	220	0.52 (-0.48, 1.52)		2.13 (-0.94, 5.21)	
**Physical activity**			0.769		0.620
Sedentary or low	3,251	0.06 (-0.17, 0.29)		1.21 (0.32, 2.10)	
Moderate or high	1,860	0.01 (-0.31, 0.32)		1.48 (0.37, 2.59)	
**Tobacco use**			0.788		0.471
Never	1,882	-0.01 (-0.30, 0.28)		1.12 (0.06, 2.18)	
Former	1,673	0.13 (-0.18, 0.44)		1.85 (0.73, 2.96)	
Current	1,556	0.06 (-0.23, 0.35)		1.29 (0.24, 2.34)	
**Alcohol consumption**			0.296		0.235
Low	1,679	-0.10 (-0.38, 0.17)		0.86 (-0.19, 1.91)	
Medium	1,720	0.20 (-0.09, 0.48)		1.85 (0.79, 2.92)	
High	1,646	0.11 (-0.19, 0.41)		1.60 (0.49, 2.72)	
**Total Food Score**			0.689		0.719
Poor	1,150	-0.02 (-0.35, 0.32)		1.00 (-0.21, 2.20)	
Inadequate	915	-0.09 (-0.47, 0.28)		1.42 (0.11, 2.71)	
Fair	1,207	0.21 (-0.12, 0.54)		2.04 (0.85, 3.23)	
Good	932	0.08 (-0.30, 0.46)		1.54 (0.24, 2.85)	
Excellent	907	0.22 (-0.19, 0.63)		1.56 (0.17, 2.96)	
**Job strain**			0.094		0.108
No	4,481	0.02 (-0.19, 0.24)		1.08 (0.22, 1.94)	
Yes	427	0.48 (-0.04, 0.99)		2.40 (0.74, 4.07)	
**Psychological distress**			0.547		0.833
Quartile 1	1,192	0.19 (-0.16, 0.53)		1.64 (0.44, 2.85)	
Quartile 2	1,286	0.15 (-0.18, 0.48)		1.18 (-0.01, 2.38)	
Quartile 3	1,608	0.09 (-0.19, 0.38)		1.58 (0.49, 2.67)	
Quartile 4	1,025	-0.15 (-0.51, 0.21)		1.09 (-0.17, 2.35)	
**Sleep disturbances**			0.861		0.577
No	3,672	0.04 (-0.18, 0.26)		1.26 (0.37, 2.14)	
Yes	1,438	0.07 (-0.24, 0.39)		1.57 (0.45, 2.68)	

**Predictors**[a]	**N**	**BMI**[b]	**p-value**[c]	**Waist circumference**[b]	**p-value**[c]
**Annoyance aircraft noise**			0.327		0.216
No	4,483	-0.01 (-0.22, 0.21)		1.17 (0.28, 2.05)	
Yes	618	0.19 (-0.15, 0.52)		1.93 (0.73, 3.13)	
**Annoyance other noise**			0.279		0.973
No	3,825	0.12 (-0.10, 0.33)		1.37 (0.50, 2.25)	
Yes	1,283	-0.08 (-0.40, 2.41)		1.35 (0.22, 2.48)	
**Changing home address**			0.680		0.096
No	3,699	0.15 (-0.36, 0.66)		2.64 (0.96, 4.31)	
Yes	1,407	0.07 (-0.16, 0.30)		1.69 (0.80, 2.58)	

[a]All covariates were classified according to baseline-values except noise annoyance and changing home address which were assessed at follow-up. [b]Random effects linear regression model adjusted for sex, age, family history of diabetes, socioeconomic status, physical activity, tobacco use and psychological distress on individual-level as well as mean income (yearly) and unemployment (%) on area-level. [c]P-values were assessed by a Wald-test for overall interaction. Alpha = 0.10.

Table S2. Associations [OR (95% CI)] of aircraft noise [≥50 versus <50)] with prediabetes, Type 2 diabetes, and both outcomes combined according to potential effect modifiers (N=5,156).

**Predictors**[a]	**n cases**	**Prediabetes**[b]	**p-value**[c]	**n cases**	**Type 2 diabetes**[b]	**p-value**[c]	**n cases**	**Combined**[b]	**p-value**[c]
**Sex**			0.557			0.053			0.130
Men	236	0.80 (0.52, 1.22)		102	0.70 (0.38, 1.30)		338	0.73 (0.50, 1.08)	
Women	176	0.96 (0.59, 1.58)		57	1.68 (0.85, 3.31)		233	1.11 (0.72, 1.69)	
**Age**[d]			0.883			0.423			0.768
<47	139	0.83 (0.49, 1.42)		49	1.25 (0.59, 2.62)		188	0.92 (0.58, 1.46)	
≥47	273	0.88 (0.58, 1.31)		110	0.86 (0.48, 1.54)		383	0.84 (0.59, 1.22)	
**Family history of diabetes**			0.376			0.507			0.220
Negative	142	0.69 (0.38, 1.26)		35	0.72 (0.24, 2.10)		177	0.66 (0.38, 1.14)	
Positive	270	0.95 (0.64, 1.39)		124	1.06 (0.63, 1.78)		394	0.97 (0.69, 1.37)	
**Physical activity**			0.024			0.532			0.020
Sedentary or low	296	1.05 (0.73, 1.51)		117	1.08 (0.64, 1.82)		413	1.04 (0.75, 1.45)	
Moderate or high	116	0.37 (0.16, 0.87)		42	0.77 (0.29, 2.03)		158	0.45 (0.23, 0.87)	
**Sleep disturbances**			0.822			0.941			0.828
No	295	0.88 (0.60, 1.29)		107	1.00 (0.57, 1.75)		402	0.89 (0.63, 1.26)	
Yes	117	0.81 (0.44, 1.49)		52	0.96 (0.43, 2.17)		169	0.83 (0.50, 1.40)	
**Annoyance aircraft noise**			0.295			0.553			0.187
No	363	0.96 (0.65, 1.41)		138	1.07 (0.62, 1.87)		501	0.97 (0.69, 1.38)	
Yes	49	0.64 (0.33, 1.25)		21	0.78 (0.31, 1.98)		70	0.63 (0.35, 1.12)	
**Changing home address**			0.070			0.633			0.237
No	307	2.17 (0.78, 6.02)		107	0.73 (0.19, 2,80)		414	1.40 (0.61, 3.24)	
Yes	105	1.01 (0.70, 1.46)		51	0.92 (0.52, 1.64)		156	0.96 (0.68, 1.35)	

[a]All covariates were classified according to baseline-values except aircraft noise annoyance and changing home address which were assessed at follow-up. [b]Random effects logistic regression model adjusted for sex, age, family history of diabetes, socioeconomic status, physical activity, tobacco use and psychological distress on individual-level as well as mean income (yearly) and unemployment (%) on area-level. [c]P-values were assessed by a Wald-test for overall interaction. Alpha = 0.10. [d]According to the median age at baseline in the study population.
